# Cost-effectiveness of an integrated 'fast track' rehabilitation service for multi-trauma patients: A non-randomized clinical trial in the Netherlands

**DOI:** 10.1371/journal.pone.0213980

**Published:** 2019-03-22

**Authors:** Ben F. M. Wijnen, Bea Hemmen, Ans I. E. Bouman, Henk van de Meent, Ton Ambergen, Peter R. G. Brink, Henk A. M. Seelen, Silvia M. A. A. Evers

**Affiliations:** 1 Center for Economic Evaluation, Trimbos Institute, Netherlands Institute of Mental Health and Addiction, Utrecht, the Netherlands; 2 Department of Clinical Epidemiology and Medical Technology Assessment, Maastricht University Medical Centre, Maastricht, the Netherlands; 3 Adelante Center of Expertise in Rehabilitation and Audiology, Hoensbroek, the Netherlands; 4 Department of Rehabilitation Medicine, Faculty of Health, Medicine and Life Sciences, Caphri, Care and Public Health Research Institute, Maastricht University, Maastricht, the Netherlands; 5 Department of Health Services Research, Faculty of Health, Medicine and Life Sciences, Caphri, Care and Public Health Research Institute, Maastricht University, Maastricht, the Netherlands; 6 Department of Rehabilitation, Radboud University Nijmegen Medical Center, Nijmegen, the Netherlands; 7 Department of Methodology and Statistics, Faculty of Health, Medicine and Life Sciences, Caphri, Care and Public Health Research Institute, Maastricht University, Maastricht, the Netherlands; 8 Netwerk Acute Zorg Limburg, Maastricht University Medical Center, Maastricht, the Netherlands; IRCCS E. Medea, ITALY

## Abstract

**Background:**

Multidisciplinary rehabilitation has been recommended for multi-trauma patients, but there is only low-quality evidence to support its use with these patients. This study examined whether a Supported Fast track multi-Trauma Rehabilitation Service (Fast Track) was cost-effective compared to conventional trauma rehabilitation service (Care As Usual) in patients with multi-trauma from a societal perspective with a one-year follow-up.

**Methods:**

An economic evaluation alongside a prospective, multi-center, non-randomized, controlled clinical study, was conducted in the Netherlands. The primary outcome measure was the Functional Independence Measure (FIM). Generic Quality of Life and Quality Adjusted Life Years (QALYs) of the patients were derived using the Short-form 36 Health Status Questionnaire.

Incremental Cost-Effectiveness Ratios (ICERs) were stated in terms of costs per unit of FIM improvement and costs per QALY. To investigate the uncertainty around the ICERs, non-parametric bootstrapping was used.

**Results:**

In total, 132 patients participated, 65 Fast Track patients and 67 Care As Usual patients. Mean total costs per person were €18,918 higher in the Fast Track group than in the Care As Usual group. Average incremental effects on the FIM were 3.7 points (in favor of the Fast Track group) and the incremental (extra) bootstrapped costs were €19,033, resulting in an ICER for cost per FIM improvement of €5,177. Care As Usual dominated Fast Track in cost per QALY as it gave both higher QALYs and lower costs. All sensitivity analyses attested to the robustness of our results.

**Conclusions:**

This study demonstrated that a multidisciplinary rehabilitation program for multi-trauma patients according to the supported fast track principle is promising but cost-effectiveness evidence remains inconclusive. In terms of functional outcome, Fast Track was more expensive but yielded also more effects compared to the Care As Usual group. Looking at the costs per QALYs, unfavorable ICERs were found. Given the lack of a willingness-to-pay threshold for functional recovery and the relatively short time horizon, it is not possible to draw firm conclusions about the first.

**Trial registration:**

(Current Controlled Trials register: ISRCTN68246661).

## Introduction

In the Netherlands, around 2,500 multi-trauma patients are treated each year [1]. Although these patients constitute a small proportion of hospitalized trauma patients in the Netherlands, they often suffer from sequelae and need long-term rehabilitation. Moreover, estimates of the medical costs and economic production losses to society clearly demonstrate that trauma should be a major concern for health policy makers and the medical profession [2]. Multidisciplinary rehabilitation has been recommended for multi-trauma patients to support multidisciplinary intervention in this population [3]. An expanding body of evidence for the (cost-)effectiveness of multidisciplinary rehabilitation in other conditions, particularly for stroke or ‘stroke units’, is available [4–7]. Recently, however, a study based on the national UK Rehabilitation Outcomes Collaborative (UKROC) database demonstrated the cost efficiency of rehabilitation for medically unstable patients with complex rehabilitation needs, and showed that rehabilitation can provide value for money by reducing on-going care costs, especially in highly dependent patients [8]. In addition, a study based on the same database, focusing on specialist inpatient multidisciplinary rehabilitation for working-aged adults, also demonstrated promising results [9].

Early multidisciplinary rehabilitation can lead to reduced stay in hospital, earlier functional gains and improved rates of home discharge once patients are fit to engage in a rehabilitation program [10]. Hence, a new rehabilitation approach was developed integrating and coordinating the treatment of multi-trauma patients between the trauma surgeon and the rehabilitation physician from an early stage post-trauma. Conceptually, an analogy exists between this approach and that of ‘stroke units’. The program, called ‘Supported Fast track multi-Trauma Rehabilitation Service’ (Fast Track = FT), has been contrasted with the conventional multi-trauma care service in an non-randomized-controlled trial (Care as Usual = CAU).[11] Both programs were found to be effective, i.e. both resulted in improved functional health status and quality of life of patients [12]. In addition, a faster (maximum) recovery in functional status was observed for FT at 6 months compared to 9 months for CAU.

The present article describes the economic evaluation, which was an integral part of the clinical study. The research question was whether a new rehabilitation service for multi-trauma patients (FT), when compared to conventional trauma rehabilitation care (CAU), would be preferable in terms of costs, effects and utilities from a societal perspective. The hypothesis was that FT is associated with a reduction in health care costs, patients’ costs and an improvement in quality of life when compared to CAU. FT was expected to be cost-effective.

## Material and methods

### Design

A prospective, multi-center, non-randomized, controlled clinical study, the ‘Supported Fast track multi-Trauma Rehabilitation Service’, was conducted in the Netherlands between 2009 and 2012, combined with an economic evaluation from a societal perspective with a time horizon of one year. Details of the study design and the effects on health related measures have been published elsewhere [11, 12]. Ethical approval for the study was obtained from the Medical Ethics Committee of Adelante Rehabilitation Center, Hoensbroek, the Netherlands. The participants provided written informed consent. The study is listed in the Current Controlled Trials (ISRCTN68246661).

### Participants

The study was conducted in three (academic) hospitals, namely Zuyderland Hospital, location Heerlen (formally known as Atrium Medical Center Heerlen), Maastricht University Medical Center and Radboud University Nijmegen Medical Center, and three rehabilitation centers, namely Adelante Rehabilitation Center, the Sint Maartenskliniek and Rehabilitation Medical Center Groot Klimmendaal. Multi-trauma patients admitted to one of the A&E departments of the participating hospitals were assessed for eligibility. Inclusion criteria were: age 18 years or older; hospitalization; a rehabilitation indication (expectation of lasting impairments or handicaps); and adequate Dutch language skills. Exclusion criteria were alcohol and/or drug abuse or severe psychiatric problems. In the study, multi-trauma was defined as having at least two or more injuries of which at least one is life threatening, including a) trauma with an Injury Severity Scale score (ISS) ≥16, b) complex multiple injuries on both lower extremities, c) a combination of one upper and one lower extremity injury, the latter of which cannot be used for load-bearing, or d) complex pelvis/acetabulum fractures. Eligible patients from Network Acute Care Limburg (in the south of the Netherlands) were included in the *FT* group and eligible patients from the Acute Care region East (in the east of the Netherlands) in the *CAU* group.

### Rehabilitation program

The program for the *intervention* FT group consisted of an integrated multi-trauma rehabilitation service approach, featuring: shorter stay in hospital and earlier transfer of multi-trauma patients to a specialized trauma rehabilitation unit; an earlier start of both specific ‘non-weight bearing’ rehabilitation training and multidisciplinary treatment; early individual goal setting; an integrated co-ordination of treatment between trauma surgeon and rehabilitation physician; and a shorter stay in the trauma rehabilitation unit. The CAU group patients received the conventional multi-trauma care service in the Netherlands, that is: the patients are admitted to hospital via the A&E. After possible surgery, they are transferred to the Intensive Care unit (IC), followed by the general surgery ward, where the patient may stay for several days/weeks. The trauma surgeon will only seek the advice of the rehabilitation physician if necessary. Esuing treatment takes place in a hospital’s outpatient clinic, in a (more distant) rehabilitation center, in a nursing home or with a local general practitioner (GP) or community physiotherapist. Typically, each of the CAU ‘stages’ may have its own more-or-less autonomous treatment perspective, depending on the professional’s individual treatment views and experience [13].

### Patient outcomes

The primary outcome measure for the cost-effectiveness analysis (CEA) was the Functional Independence Measure (FIM), a measure for functional health status [14]. Both generic Quality of Life and utilities were derived from the Short-form 36 Health Status Questionnaire (SF-36). The SF-36 gives a profile for a particular health status. For the SF-6D utilities an overall utility score for population-based quality of life was obtained, which facilitates comparisons with other interventions, that is, the social tariff of the SF-36 [15, 16]. For our study we used an algorithm established using a general population from the UK. This algorithm converts health states into utilities. The primary outcome measure for the cost-utility analysis (CUA) was utilities based on the SF-6D [17]. Utilities represent preferences for different health states and allowed quality-adjusted life years (QALYs) to be calculated by multiplying the overall utility score with lifespan. As the time horizon of the CUA presented here was one year, the utility values (multiplied by one) equaled the QALYs for each patient (provided that the patient was still alive at that time, so no mortality occurred). The primary outcomes were measured by questionnaires at baseline and at 3, 6, 9, and 12 months post-trauma.

### Costs

The following costs were considered: a) health care costs: (*in-patient*) length of stay at the hospital/IC-unit, day treatments, contacts with medical specialists and paramedics, length of stay at the rehabilitation center, rehabilitation therapy, and length of stay at the nursing home or home for the elderly; (*outpatient*) rehabilitation therapy, contacts with medical specialists, GPs and paramedics, home care help and medication; (*intervention program*) no separate cost was calculated as the FT program had been part of the daily operation of a specialized rehabilitation clinic since 2006; b) patient & family costs: informal care, paid domestic help, over-the-counter medication, aids and in-home modifications; c) and other costs: (*for patients in paid employment*) production losses to society due to absenteeism (illness-related absence from work), presenteeism (loss of productivity while at work), and compensation mechanisms. Diminished productivity due to absence from work may be compensated when lost work can be made up by the sick employees themselves or taken over by other employees within the company during normal working hours.

The measures for health care cost *volumes* were obtained from formal registers of three participating hospitals and three rehabilitation centers, and/or were recorded in a cost questionnaire. The measures of the volumes of the patient & family costs were also recorded in the cost questionnaire. Production losses were measured using the patient modules of the Productivity and Disease Questionnaire (PRODISQ) [18]. The PRODISQ was used together with the cost questionnaire at baseline and at 3, 6, 9, and 12 months post-trauma, measuring resource use with a 3-month recall period. The baseline questionnaire gave the volumes of the pre-trauma period to determine any differences between the groups at the start of the follow-up period. For the *valuation* of health care costs and patient & family costs, an update of the Dutch manual for cost-analysis in health care research was used [19]. Medication was valued according to the average cost per prescription drug, including the pharmacists’ prescription fee [20]. For care without available cost guidelines, average real costs were used. Where possible, costs were participant-specific. The value of lost productivity was calculated applying the Friction Cost Approach with a friction period of 23 weeks. This method assumes that productivity losses only occur during the ‘friction period’; this period reflects the time needed to replace (and train) a new worker [21].

All costs were indexed to the year 2016 (in Euros).

### Economic analysis

For the CEA, we calculated the incremental cost and effectiveness of the FT program compared with conventional multi-trauma care. Incremental costs are defined as the mean difference between both groups in total costs over 12 months. Incremental effectiveness is the mean difference in the FIM scores over 12 months. For the CUA, the incremental cost-utility was calculated as the difference in total costs divided by the difference in QALYs. The Incremental Cost-Effectiveness Ratios (ICERs) were given as costs (€) per unit improvement in the FIM and costs (€) per QALY.

All analyses were performed according to the intention-to-treat principle. Clinical differences between the FT and CAU group, were assessed using a linear mixed-effects regression model in SPSS, version 20.0 (SPSS, Inc., Chicago, IL). More information on the clinical effectiveness evaluation can be found in Bouman et al. [12]. Missing data were imputed by extrapolation (in case of partial missing values, e.g., some follow-up data present) or by the overall mean for the respective variable per study group (in case of complete missings).

As cost data is generally skewed and not distributed normally, non-parametric bootstrap re-sampling techniques were performed in STATA 14, with 5,000 replications to estimate cost-effectiveness uncertainty intervals around the ICERs [22, 23]. Bootstrapping is a non-parametric way to repeatedly conduct an analysis by resampling, with replacement, from the observed data [24]. Seemingly unrelated regression equations (SURE) were bootstrapped (5000 times) to allow for correlated residuals of the cost and utility equations. The uncertainty interval is represented by the 2.5^th^ and 97.5^th^ percentiles. The results of ICER bootstraps are presented in cost-effectiveness planes and cost-effectiveness acceptability curves (CEACs)[25]. Cost-effectiveness planes show differences in effect on the horizontal axis and costs on the vertical axis. Bootstrapped cost-effectiveness pairs located in the northwest quadrant indicate the FT to be inferior to conventional care (more costly and less effective); in the southeast quadrant to be dominant (more effective and less costly); and with respect to the north-east and south-west quadrant, the preference for an intervention depends on the threshold value, that is, what society is prepared to pay for an effectiveness gain, or willing to accept as savings for effectiveness loss. The CEAC represents the probability that, given a certain threshold for the willingness to pay for an extra point on the FIM or for a QALY, the intervention is cost-effective. A CEAC is constructed by taking certain thresholds (€) and calculating the percentage of the 5000 bootstrapped ICERs that are below each threshold, and therefore cost-effective, given that threshold. In the Netherlands, thresholds may vary between €18,000 to €80,000 per QALY depending on the burden of disease [26]. This results in a curve with thresholds on the x-axis and probability of the intervention being cost-effective on the y-axis.

Finally, a number of sensitivity analyses were performed. First, the Human Capital method was used to value lost productivity instead of the Friction Cost Approach. This method counts any hour not worked as an hour lost (at €35.77). Second, analyses were performed from a healthcare perspective which excluded all patient & family costs and productivity losses. This was done as the healthcare perspective is still a dominant perspective in health economics and is recommended as main perspective in certain countries—for example, in the United Kingdom by The National Institute for Health and Care Excellence [27]. And third, a subgroup analysis including only patients who were admitted to a rehabilitation center for inpatient care was used instead of the total group. Lastly, a sensitivity analysis was conducted in which a correction was made for baseline FIM scores.

## Results

### Participants

**Fig 1:**
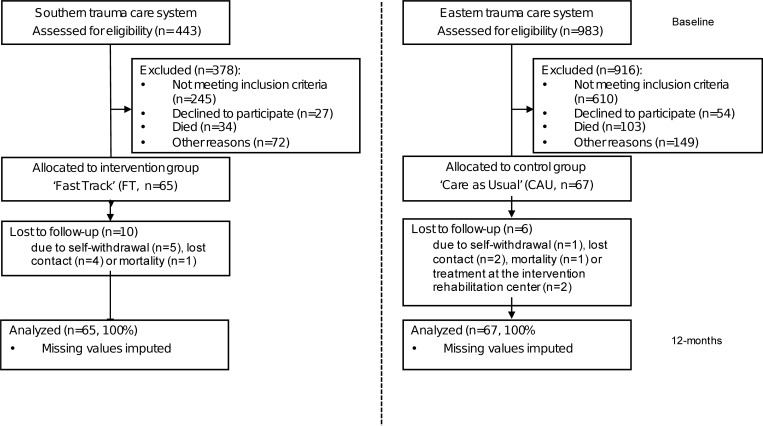
Flow Diagram of the participants (as published in [12]).

In total, 132 patients participated: 65 FT patients from Network Acute Care Limburg (in the south of the Netherlands) and 67 CAU patients from Acute Care region East (in the east of the Netherlands). Baseline characteristics for most variables were comparable between the groups (Table 1, as published in [12]). Volumes of cost items during the 3-month pre-trauma period were low and comparable between the groups (Table 2). A flow diagram of the participants is shown in Fig 1.

**Table 1:** Baseline characteristics of the participants.

Characteristic^a^	Sample size (FT/CAU)	Fast Track (n = 65)	Care as Usual (n = 67)	P-value^b^
Age at injury, mean (SD)	65/67	44.7 (16.7)	42.0 (16.6)	0.34^1^
Range		18–75	18–73	
Gender, Male	65/67	49 (75)	56 (84)	0.24^2^
Marital status	64/63			0.54^3^
Married/living together		32 (50)	38 (60)	
Divorced/widowed		12 (19)	5 (8)	
Single		20 (31)	20 (32)	
Education	65/65			0.20^3^
Primary school / lower (professional) education		13 (32)	15 (40)	
Middle (professional) education		16 (40)	16 (42)	
Higher (professional) education		11 (28)	7 (18)	
Informal care, Yes	64/65	51 (80)	58 (89)	0.13^2^
Pre-trauma health disorders, Yes	63/66	45 (71)	39 (59)	0.14^2^
Pre-trauma work status, Employed	62/62	39 (63)	40 (64)	0.85^2^
Type of accident	65/64			0.77^3^
Traffic accident		41 (63)	39 (61)	
Fall		15 (23)	15 (23)	
Other		9 (14)	10 (16)	
Type of injury	65/67			0.39^3^
Multi-trauma (neuro-trauma and musculoskeletal injuries)		14 (22)	33 (49)	
Musculoskeletal injuries only		48 (74)	28 (42)	
Neuro-trauma		3 (5)	6 (9)	
ISS, score 0–75, mean (SD)	64/67	22.1 (12.8)	29.4 (11.2)	<0.001^1^
Range		4–66	4–50	
Median (IQR)		19.5 (12–29)	29 (21–38)	
Complications during hospital stay, Yes	61/66	19 (31)	37 (56)	0.01^2^
MMSE, score 0–30, mean (SD)^c^	58/47	26.6 (4.4)	26.9 (3.4)	0.69^4^
FIM, score 18–126, mean (SD)^d^	55/60	89.3 (25.0)	93.9 (32.9)	0.40^1^
SF-36, score 0–100, mean (SD)	37/40	89 (8.8)	86 (12.9)	0.17^1^
HADS, 0–42, mean (SD)	55/51	11.7 (8.8)	12.0 (8.2)	0.86^1^

CAU = Care as Usual, FT = Fast Track, IQR = interquartile range, ISS = Injury Severity Score, MMSE = Mini-Mental State Examination, SD = standard deviation.

^a^ Values are numbers (percentages) unless stated otherwise.

^b^ Significant *p*-value set at 0.05 (two-tailed): 1) independent sample *t*-test, 2) Pearson’s Chi-square test, 3) one-way ANOVA, 4) Mann-Whitney U-test.

^c^ Scores of 25 or higher are being considered normal cognitive functioning. A number of patients were not able to perform the test due to injury severity.

^d^ Baseline value of the outcome measure for participants included in the sensitivity analysis in which a baseline correction was performed

**Table 2:** Volumes of cost items during the 3-month pre-trauma period.

		Fast Track (n = 65)				Care as usual (n = 67)			
Variable	Sample size (I/C)	Subjects n (%)	Mean	SE	Min-Max	Subjects n (%)	Mean	SE	Min-Max
Hospital									
Admission	64/64	0 (0)				1 (2)			
Medical specialist consultation, n	64/64	16 (25)	0.52	0.178	0–10	14 (22)	0.30	0.082	0–3
GP consultation, n	63/64	20 (32)	0.59	0.141	0–6	17 (27)	0.41	0.099	0–4
Paramedical care*, n (consultation)	51/64	4 (8)	0.55	0.312	0–12	2 (3)	0.27	0.202	0–12
Home care use^†^, hours	64/61	3 (5)	2.25	1.306	0–60	1 (2)	0.59	0.590	0–36
Informal care, hours	63/62	3 (5)	0.76	0.460	0–24	1 (2)	2.32	2.323	0–144
Medication, n (prescription drug)	60/63	25 (42)	1.08	0.223	0–9	15 (24)	0.81	0.248	0–9
Aids	64/63	8 (12)				4 (6)			
In-home modification	64/63	0 (0)				1 (2)			

GP = general practitioner; n = number; SE = standard error

* Pre-trauma measurement is reported for physiotherapy only (mainly used)

† Only practical assistance at home was reported for the pre-trauma period.

In the intervention group 10 (FT) patients (out of 65, 15%) and in the control group (CAU) 6 patients (out of 67, 9%) were lost to follow-up. The reasons for these drop-outs included self-withdrawal (FT/CAU, 5/6), lost contact (FT/CAU, 4/1) and mortality (FT/CAU, 1/1). At baseline, the differences in FIM and SF-36 scores were not statistically significant between the groups. For the SF-36, the baseline values represented pre-trauma measurements. Results of the linear mixed-effects regression model demonstrated that, although patients in both groups improved their functional health status and quality of life, there were, in general, no differences in effectiveness between the groups at the 12-month follow-up. Also, a faster (maximum) recovery in functional status was observed for Fast Track at 6 months compared to 9 months for Care as Usual [12]. The utility scores derived from the SF-36 are presented in Table 3; the differences between the groups at 3, 6, 9 and 12 months post-trauma are not statistically significant. Furthermore, no differences in QALY’s between the groups were found.

**Table 3:** Utility scores, QALYs, and FIM scores (uncorrected).

		Fast Track group (n = 55)		Care as usual group (n = 61)		
**Mean utility per time point**		Mean	SD	Mean	SD	P-value*
	3 months	0.63	0.11	0.65	0.09	0.43
	6 months	0.68	0.12	0.69	0.09	0.25
	9 months	0.69	0.11	0.68	0.11	0.47
	12 months	0.69	0.11	0.70	0.11	0.58
						Difference^†^
**QALY**^‡^		0.67		0.68		-0.011 (-0.041 to 0.018)
**Mean FIM score per time point**			SD		SD	
	3 months	115.20	12.25	116.22	13.77	0.65
	6 months	119.23	8.49	118.28	12.80	0.62
	9 months	120.73	7.09	120.10	11.11	0.70
	12 months	120.07	7.70	120.86	11.90	0.65

QALY = quality adjusted life years; SD = standard deviation; SE = standard error

* Mann-Whitney U test for utilities, independent t-test for FIM scores

† Bootstrapped 1,000 times (2.5^th^—97.5^th^ percentile)

‡ Equals (0.25*T3) + (0.25*mean T3, T6) + (0.25*mean T6,T9) + (0.25*mean T9,T12) utility scores.

### Costs

Valuations of cost items are shown in Table 4. A detailed overview of resource use for the 12-months follow-up can be found in S1 Table. The FT group received, on average, more rehabilitation in terms of length of stay (days) and treatment (hours) than the CAU group. This was mostly due to a higher percentage of the Fast Track group admitted to a rehabilitation center (77% versus CAU 58%) and a higher percentage receiving outpatient rehabilitation treatment (80% versus CAU 40%) (not tabulated). This was contrary to our expectations. The CAU group spent, on average, more days at the hospital and IC, and received more community physiotherapy and informal care than the FT group, as expected.

**Table 4:** Valuation of cost items during the 12-month follow-up period (in Euro; 2016).

Category	Volume	Cost*	Source of Data^†^
*Health care*: *inpatient*			
Hospital			
LOS^§^	day	648	Local hospital Questionnaire^‡^
IC-unit	day	2,034	
Day treatment	day	165	
Rehabilitation center			
LOS	day	464	Rehabilitation center Questionnaire^‡^
Rehabilitation therapy^¶^	hour	154	
Nursing home	day	170	Questionnaire
*Health care*: *outpatient*			
Rehabilitation center			
Rehabilitation therapy^¶^	hour	154	Rehabilitation center Questionnaire^‡^
Medical specialist	consultation	168	Local hospital Questionnaire^‡^
	telephone consultation	82	
GP	home visit	33	Questionnaire
	consultation	50	
	telephone consultation	17	
Paramedical care^ǁ^			
Physiotherapy	consultation	33	Questionnaire
Occupational therapy	consultation /hour	33	
Speech therapy	consultation	30	
Social work	consultation	66	
Other	consultation /hour	43	
Practical assistance at home	hour	23	
Personal care at home^#^	hour	50	
Medication (prescription drug) ^††^	number	28	
*Patient and family*			
Informal care**	hour	14	Questionnaire
Medication (over the counter)^††^	number	8	
Aids^§§^	number	total	
In-home modifications^§§^	number	total	
*Other cost*			
Production losses^¶¶^			Questionnaire

GP = general practitioner; IC = intensive care; LOS = length of stay

* Prices (2016 €) from the Dutch manual for cost-analysis in health care research, unless otherwise stated. Prices are for academic hospitals

† Continuous formal registration over 12 months from three local hospitals and three rehabilitation centers participating in the research project. Data from questionnaires at 0, 3, 6, 9, and 12 months were used for admissions/treatment in other hospitals and/or rehabilitation centers, and for remaining variables

‡ Complementary data for other (non-local) hospital admissions, outpatient medical specialist consultations, and treatment at other rehabilitation centers

§ The cost for medical specialist consults, paramedical care and medication is included in the hospital day price

¶ Rehabilitation center: cognitive training, physiotherapy, occupational therapy, speech therapy, social work, psychologist, physiatrist, and other

ǁ Community care: physiotherapy, exercise/activities therapy, occupational therapy, speech therapy, social work, and other (self-reported, e.g., psychologist, manual therapy, hydrotherapy, and dietary advice; the price per consultation/hour is an estimated average)

# No distinction was made in the questionnaire between personal care and nursing care; the reported price is for personal care (the price for nursing care is €65 per hour)

** The cost for informal care is in accordance with the standard hour tariff for cleaning work

†† Number of medications at 3, 6, 9 and 12 months; the average cost per prescription drug includes the pharmacist prescription fee (€7.28), and the over-the-counter medication is valued according to (average) real costs

§§ Number of acquired aids and in-home modifications over 12 months; aids are valued according to (average) real costs and the cost for in-home modifications are self-reported

¶¶ Production losses are valued according to the Friction Cost Approach.

The total mean costs per person were €18,918 higher in the FT group than in the CAU group (see Table 5). The difference between the groups was mainly caused by the higher cost for rehabilitation in the FT group. Although higher costs in the CAU group were incurred by length of stay at the hospital and IC, community physiotherapy and informal care, these costs did not outweigh the higher costs for rehabilitation in the FT group. The cost for production losses due to absence from work was comparable for both groups. The percentage of patients in paid employment who returned to work in the first year after trauma was, respectively, 38% and 52% for the FT and CAU group; the mean time back to work was, respectively, 5.1 and 5.7 months (not tabulated). Subsequently, most patients were absent from work during the friction period of 23 weeks, and calculated costs for production losses were, on average, comparable for both groups.

**Table 5:** Costs (in Euro; 2016) over the 12-month follow-up period.

		FT group (n = 65)	CAU group (n = 67)
Variable	Cost per unit^†^ (€)	Cost* (€)	Cost* (€)
*Health care*: *inpatient*			
LOS hospital	648/day	16,375	16,858
IC-unit	2034/day	7,910	9,884
Day treatment	165/day	76	46
LOS rehabilitation center	464/day	21,011	16,530
Rehabilitation therapy	154/hour	12,262	7,659
Nursing home	170/day	216	886
*Health care*: *outpatient*			
Rehabilitation therapy	154/hour	15,121	3,035
Medical specialist	163/contact	1,050	1,093
telephone	82/contact	6	6
GP	33/contact	95	115
home visit	50/contact	41	49
telephone	17/contact	13	23
Paramedical care			
Physiotherapy	33/contact	1,410	1,590
Occupational therapy	33/contact	212	173
Speech therapy	30/contact	37	147
Social work	66/contact	70	118
Other	43/contact	187	389
Home care			
Practical assistance	23/hour	524	342
Personal care	50/hour	58	232
Medication (prescription)	28/drug	231	170
*Patient and family*			
Informal care	14/hour	2,110	2,856
Medication (over counter)	8/drug	10	13
Aids	mean total	660	405
In-home modification	mean total	592	50
*Other cost*			
Production losses^§^		15,545	15,089
TOTAL COST (95% CI)		93,786 (81,191–108,022)	74,868 (62,831–86,624)

CI = confidence interval; GP = general practitioner; IC = intensive care; LOS = length of stay

* Mean cost over the entire study group (12 months)

^†^ Separation of cost for the Fast Track program was not applicable

^§^ Friction Cost Method (maximum 23/52 weeks absent = 0.4423 years *1540 hours * €32.25

### Cost-effectiveness

**Fig 2:**
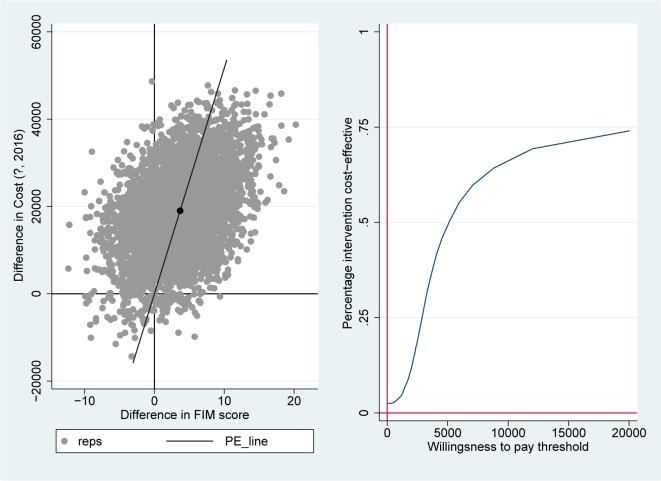
Incremental cost-effectiveness plane (left) and cost-effectiveness acceptability curve (right) for cost per point improvement on the FIM, showing 5000 bootstrapped cost-effectiveness pairs. PE-line: line which represents the point estimate of the ICER (average cost/effect of bootstrap replications).

**Fig 3:**
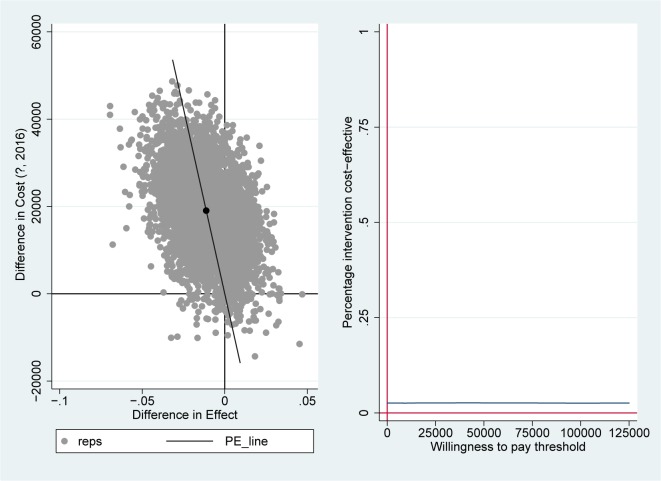
Incremental cost-effectiveness plane (left) and cost-effectiveness acceptability curve (right) for cost per QALY, showing 5000 bootstrapped cost-effectiveness pairs. PE-line: line which represents the point estimate of the ICER (average cost/effect of bootstrap replications).

The bootstrapped incremental effectiveness on the FIM was 3.7 points (in favor of the FT group) and the incremental bootstrapped (extra) costs were €19,034, resulting in an ICER for cost per improvement on the FIM of €5,177. Most of the cost-effectiveness pairs are located in the north-east quadrant (78%), where the FT program is more effective than the CAU program, but also more costly (Fig 2). In the north-west (inferior) quadrant, where the FT program is less effective and more expensive, 20% of the pairs are located. For cost per QALY, the ICER of the FT versus CAU was negative (and very high) due to an incremental effectiveness of -0.01 in favor of the CAU group. In other words, FT was dominated by CAU. The cost-effectiveness plane for the QALYs showed that 22% of the cost-effectiveness pairs are located in the north-east quadrant and 76% in the north-west (inferior) quadrant (Fig 3).

The CEAC for cost per point improvement on the FIM showed that there is a 73% chance that the FT rehabilitation is more cost-effective, given a border value of €20,000. For cost per QALY, there was only a 4% chance that the FT rehabilitation program is more cost-effective than the CAU rehabilitation (see Fig 3).

### Sensitivity analyses

Using the Human Capital Approach to value productivity losses resulted in an incremental costs difference of €21,151 for the FT group compared to CAU. The resulting ICER was €5,745 per improvement on the FIM. In terms of cost per QALY gained, similar to the base case, FT was dominated by CAU. From a healthcare perspective, an ICER of 6,632 per improvement on the FIM was found. Regarding cost per QALY gained, FT was dominated by CAU. Next, by including only patients who were admitted to a rehabilitation center for in-patient care, the FT was dominated by CAU in both cost per improvement on FIM and costs per QALY gained. Lastly, a baseline correction for baseline FIM scores also resulted in similar results (FT was dominated by CAU in both cost per improvement on FIM and costs per QALY gained).

## Discussion

The main goal of this study was to determine whether fast track care was preferable in terms of costs, effects and utilities, from a societal perspective, compared to care as usual. As previously demonstrated, both groups of multi-trauma patients improved in functional status and quality of life [12]. However, no significant differences were found between the FT and the CAU patients at 12 months post-trauma, indicating that there was no added effect of treatment over time but a faster (maximum) recovery in functional status was observed for FT at 6 months compared to 9 months CAU. Total costs were higher in the FT group than in the CAU group, which is mainly explained by higher volumes of rehabilitation therapy in this group. In terms of cost per improvement in FIM score, the FT group showed slightly higher effects but also higher costs, resulting in an ICER of €5,177 per improvement on the FIM. However, in the absence of a willingness-to-pay threshold for such a clinical measure, no statements regarding its cost-effectiveness can be made. Looking at quality of life, the FT group had lower QALYs than the CAU and higher annual costs, resulting in a dominated ICER. This would therefore indicate that FT is not cost-effective.

As shown by the sensitivity analyses, our results were not heavily affected by specific assumptions, perspectives or inclusion criteria.

There may be several reasons for the lack of added effects of FT. For example, it is likely that CAU is already provided at a high level and, consequently, it requires more effort to gain minor improvements in quality of life. This may be strengthened by the fact that quality of life is a broad concept which is only affected to a certain extent by clinical symptoms. Moreover, in a study by Engel et al. (2014), the measurement properties of the SF-6D were examined in a sample of individuals living with spinal cord injuries. They argued that the SD-6D failed to detect self-reported and clinically important health changes and emphasized that the use of the SF-6D in similar patients requires some consideration [28]. In addition, it has been argued that the SF-36 (a more extensive form than the SF-6D) captures only a small proportion of health outcomes related to trauma [29]. In support of this, a study of Denehy et al. [30] showed no differences in quality of life at 12 months after intensive exercises in the ICU and the ward and as outpatients of patients at the ICU including trauma patients using the SF-36.

Alternatively, it could be that the FT program did not differ sufficiently from the CAU. As stated by Bouman et al. (2017), more favorable results of FT may be expected after optimizing procedures [12]. In FT, the number of paramedical treatments in the rehabilitation center was considerably higher compared to CAU leading to higher costs. Reduction of costs could be achieved by looking critically at number and frequency of paramedical treatment.

Hence, as frequently argued, we believe that a multidisciplinary approach to multi-trauma patients can help optimize care, minimize morbidity and mortality, and ultimately provide a framework for accelerated post-injury rehabilitation [3, 31]. In addition, reduction of LOS in rehabilitation center could lower costs as shown by Wu et al. [32]. In the retrospective study of Wu et al. [32], the costs of transfer of multi-trauma patients to an external rehabilitation unit after acute ward compared to an ‘in-house’ rehabilitation service showed that LOS in the latter was significant lower compared to the external rehabilitation unit. Using the LOS in a rehabilitation center as a proxy for resource consumption they calculated a cost saving of $1,2 million Australian dollar per year. In contrast, in the present study, a shorter LOS was found in the CAU group and thus decreasing the total costs. Hence, it is important to critically look at the admission time in the rehabilitation center in the future.

This study is not without its limitations. First, due to the nature of the intervention, participants were not randomized, which could have introduced confounding factors which we were unable to control for. Second, the rehabilitation process focuses on increasing participation in the community and increasing functional abilities. One aspect is return to work, but individuals and society gain much more from rehabilitation. In this study, the percentage of patients in paid employment who returned to work in the first year after trauma was, respectively, 38% and 52% for the FT and CAU group; the mean time back to work was, respectively, 5.1 and 5.7 months. The present study lasted one year only, but a study duration up to two years could increase the percentages of patients who return to work possibly leading to more positive increments for the FT group. This is confirmed by another study looking at the cost-efficiency of specialist hyper acute in-patient rehabilitation services for medically instable patients including trauma patients. The authors conclude that ‘although the costs of hyper acute rehabilitation were quite high, this investment was offset by savings in the cost of on-going care within 28 months (2 years + 4 months)’ [8].

Second, given the more recently demonstrated poor performance of, in particular, the SF-6D, alternative quality of life instruments could have been used. In economic evaluations, an accurate representation of quality of life of patients is crucial as costs per QALY is important information for healthcare decision makers due to established willingness-to-pay thresholds (i.e. €20,000–80,000 per QALY for the Netherlands, depending on the severity of the disease or disorder [33]). For example, costs per QALY analyses are required by the National Institute for Health and Clinical Excellence (NICE) in the UK [34] and used by the National Health Care Institute in the Netherlands for health technology assessment [35]. However, to date, there are no suitable alternatives to measure quality of life in multi-trauma patients [29]. Third, the sample size of the study was smaller than anticipated [11]. However, the dataset upon which the original estimations were based was relatively small and the total number required may have been overestimated. Nevertheless, the study may lack the power to detect an effect of FT compared to CAU.

## Conclusions

This study demonstrated that a multidisciplinary rehabilitation program for multi-trauma patients according to the supported fast track principle is promising but cost-effectiveness evidence remains inconclusive. Although there were minor improvements in functional outcome in the FT group compared to the CAU group, no significant differences were found. Looking at the incremental economic analyses, unfavorable ICERs were found in terms of costs per QALY but, in terms of functional outcome, FT was more expensive and yielded more effects (i.e. ICER of 5,177 per improvement on FIM). However, given the lack of a willingness-to-pay threshold, it is not possible to draw firm conclusions about the latter.

Further research should aim construct or identify valid and sensitive quality of life instruments for patients with (multi-)trauma. In addition, attempts could also be made to identify factors of multidisciplinary rehabilitation programs associated with increases in quality of life in multi-trauma patients.

## Supporting information

S1 Table Volumes of cost items during the 12-month follow-up period. C = control group; CI = confidence interval; GP = general practitioner; I = intervention group; IC = intensive care; LOS = length of stay; n = number; SE = standard error; *Hospital data from formal registries were not available for 5 participants (2 I/3 C); 4 of those 5 also did not have available data from the questionnaires. Of the mean number of hospital bed days, mean 1.25 days (SE = 0.370; n = 14 C) and mean 6.12 days (SE = 1.848; n = 25 I) were attributed to hospital stay in non-participating hospitals (data from questionnaires); ^†^Of the intervention group 11 (out of 65) persons did not receive in- or outpatient rehabilitation, and of the control group 27 persons (out of 67). Two persons (out of 64) in the control group and 10 persons (out of 64) stayed in rehabilitation centres that did not participate in the project; for 5 persons in the control group rehabilitation treatment hours were not available
(DOCX)

S1 Data An anonymized version of the dataset is provided as online supplement (file: “Anonymous_dataset_Wijnen_et_al.dta”). (DTA)
