# Supplementary material for: Cost-effectiveness of an integrated 'fast track' rehabilitation service for multi-trauma patients: A non-randomized clinical trial in the Netherlands
Source: PLoS One. 2019 Mar 22;14(3):e0213980. doi: 10.1371/journal.pone.0213980 (PMC6430380; doi:10.1371/journal.pone.0213980)
Supplement: S1 Table — C = control group; CI = confidence interval; GP = general practitioner; I = intervention group; IC = intensive care; LOS = length of stay; n = number; SE = standard error; *Hospital data from formal registries were not available for 5 participants (2 I/3 C); 4 of those 5 also did not have available data from the questionnaires. Of the mean number of hospital bed days, mean 1.25 days (SE = 0.370; n = 14 C) and mean 6.12 days (SE = 1.848; n = 25 I) were attributed to hospital stay in non-participating hospitals (data from questionnaires); †Of the intervention group 11 (out of 65) persons did not receive in- or outpatient rehabilitation, and of the control group 27 persons (out of 67). Two persons (out of 64) in the control group and 10 persons (out of 64) stayed in rehabilitation centres that did not participate in the project; for 5 persons in the control group rehabilitation treatment hours were not available (DOCX) [file pone.0213980.s001.docx]

**Online supplementary material S1**

Table S1 Volumes of cost items during the 12-month follow-up period

|  |  | **Intervention group (n=65)** | | | | **Control group (n=67)** | | | |  |
| --- | --- | --- | --- | --- | --- | --- | --- | --- | --- | --- |
| **Variable** | Sample size (I/C) | Subjects  n (%) | Mean | SE | Min-Max | Subjects  n (%) | Mean | SE | Min-Max | Mean  difference |
| *Health care: inpatient* |  |  |  |  |  |  |  |  |  |  |
| Hospital*, days |  |  |  |  |  |  |  |  |  |  |
| LOS | 63/64 | 62 (100) | 25.3 | 2.19 | 8-100 | 64 (100) | 26.0 | 2.23 | 3-93 | 1.32 |
| IC-unit | 63/64 | 34 (54) | 3.89 | 0.853 | 0-27 | 42 (66) | 4.86 | 0.865 | 0-35 | 0.82 |
| Day treatment | 63/64 | 24 (38) | 0.46 | 0.090 | 0-4 | 10 (16) | 0.28 | 0.101 | 0-5 | -0.12 |
| Rehabilitation center^†^ |  |  |  |  |  |  |  |  |  |  |
| LOS, days | 64/64 | 49 (77) | 45.3 | 5.58 | 0-220 | 37 (58) | 35.6 | 5.78 | 0-211 | -11.6 |
| Rehab therapy, hours | 64/59 | 49 (77) | 79.6 | 10.70 | 0-384 | 32 (54) | 48.4 | 9.89 | 0-361 | -32.7 |
| Nursing home |  |  |  |  |  |  |  |  |  |  |
| Days | 55/61 | 1 (2) | 1.27 | 1.273 | 0-70 | 3 (5) | 5.21 | 3.109 | 0-138 | 1.23 |
| *Health care: outpatient* |  |  |  |  |  |  |  |  |  |  |
| Rehab therapy, hours | 64/62 | 51 (80) | 98.2 | 13.30 | 0-520 | 27 (40) | 19.7 | 6.36 | 0-319 | -78.4 |
| Medical specialist, n |  |  |  |  |  |  |  |  |  |  |
| Consultation | 63/66 | 60 (95) | 6.37 | 0.631 | 0-26 | 61 (92) | 6.62 | 0.619 | 0-31 | 0.45 |
| Telephone consultation | 64/66 | 12 (19) | 0.34 | 0.103 | 0-4 | 15 (23) | 0.35 | 0.107 | 0-5 | 0.01 |
| GP, n |  |  |  |  |  |  |  |  |  |  |
| Consultation | 55/61 | 34 (62) | 2.89 | 0.465 | 0-14 | 42 (69) | 3.48 | 0.619 | 0-29 | 0.42 |
| Home visit | 55/61 | 15 (27) | 0.82 | 0.311 | 0-14 | 16 (26) | 0.98 | 0.386 | 0-20 | 0.05 |
| Telephone consultation | 55/61 | 14 (26) | 0.75 | 0.244 | 0-8 | 15 (25) | 1.38 | 0.544 | 0-30 | 0.29 |
| Paramedical care, n (consultation) |  |  |  |  |  |  |  |  |  |  |
| Physiotherapy | 53/61 | 33 (69) | 42.7 | 6.90 | 0-198 | 46 (75) | 47.5 | 7.05 | 0-288 | 6.76 |
| Occupational therapy | 53/61 | 11 (21) | 6.42 | 2.000 | 0-65 | 11 (18) | 5.27 | 1.751 | 0-72 | -1.47 |
| Speech therapy | 53/61 | 1 (2) | 1.23 | 1.226 | 0-65 | 8 (13) | 5.13 | 2.282 | 0-120 | 2.83 |
| Social work | 53/61 | 8 (15) | 1.06 | 0.448 | 0-15 | 8 (13) | 1.76 | 0.740 | 0-29 | 0.86 |
| Other | 53/61 | 12 (23) | 4.34 | 2.200 | 0-108 | 21 (34) | 8.90 | 2.724 | 0-108 | 5.11 |
| Use of home care, hours |  |  |  |  |  |  |  |  |  |  |
| Practical assistance | 54/61 | 6 (11) | 22.8 | 9.54 | 0-288 | 9 (15) | 14.8 | 5.26 | 0-180 | -4.93 |
| Personal care | 54/61 | 4 (7) | 1.17 | 0.656 | 0-24 | 1 (2) | 4.64 | 4.639 | 0-283 | 0.49 |
| Medication, n (prescription) | 53/61 | 44 (83) | 8.4 | 1.10 | 0-35 | 42 (69) | 6.2 | 1.20 | 0-40 | 2.07 |
| *Patient and family* |  |  |  |  |  |  |  |  |  |  |
| Informal care, hours | 56/60 | 32 (57) | 152.1 | 36.02 | 0-1284 | 33 (55) | 206.0 | 43.28 | 0-1512 | 28.8 |
| Medication, n  (over-the-counter) | 57/61 | 30 (53) | 1.16 | 0.141 | 0-5 | 37 (61) | 1.61 | 0.167 | 0-4 | -0.52 |
| Aids, n | 57/62 | 44 (77) | 2.40 | 0.265 | 0-7 | 34 (55) | 1.03 | 0.182 | 0-7 | -1.34 |
| In-home modification, n | 58/62 | 18 (31) | 0.64 | 0.170 | 0-6 | 12 (19) | 0.26 | 0.072 | 0-2 | -0.35 |

C=control group; CI=confidence interval; GP=general practitioner; I=intervention group; IC=intensive care; LOS=length of stay; n=number; SE=standard error

* Hospital data from formal registries were not available for 5 participants (2 I/3 C); 4 of those 5 also did not have available data from the questionnaires. Of the mean number of hospital bed days, mean 1.25 days (SE=0.370; n=14 C) and mean 6.12 days (SE=1.848; n=25 I) were attributed to hospital stay in non-participating hospitals (data from questionnaires)

† Of the intervention group 11 (out of 65) persons did not receive in- or outpatient rehabilitation, and of the control group 27 persons (out of 67). Two persons (out of 64) in the control group and 10 persons (out of 64) stayed in rehabilitation centres that did not participate in the project; for 5 persons in the control group rehabilitation treatment hours were not available
